# Assessment of 4D flow MRI for quantification of left-to-right shunt in pediatric patients with ventricular septal defect: comparison with right heart catheterization

**DOI:** 10.3389/fcvm.2024.1399110

**Published:** 2024-07-22

**Authors:** Seyed Mohammad Zamani-Aliabadi, Salah D. Qanadli, Seyed Hasan Fatehi-Feyzabad, Mohsen Ghasemnezhad, Hamidreza Ghaemi, Arshid Azarine, Ali Mohammadzadeh, Ahmad Bitarafan-Rajabi, Hojjat Mortezaeian, Kiara Rezaei-Kalantari

**Affiliations:** ^1^ Department of Medical Physics, School of Medicine, Iran University of Medical Sciences, Tehran, Iran; ^2^ Cardiothoracic and Vascular Division, Department of Diagnostic and Interventional Radiology, Lausanne University Hospital and University of Lausanne, Lausanne, Switzerland; ^3^ Department of Radiology, Rajaie Cardiovascular Medical and Research Center, Iran University of Medical Sciences, Tehran, Iran; ^4^ Department of Pediatric Cardiology, Rajaie Cardiovascular Medical and Research Center, Iran University of Medical Sciences, Tehran, Iran; ^5^ Department of Radiology, Hôpital Marie Lannelongue, Groupe Hospitalier Paris Saint-Joseph, Université Paris-Saclay, Paris, France

**Keywords:** right heart catheterization, cardiac magnetic resonance imaging, four-dimensional (4D) flow, two-dimensional (2D) flow, shunt fraction, ventricular septal defect (VSD), congenital heart disease (CHD)

## Abstract

**Objectives:**

The percentage of shunt fraction significantly impacts the management of patients with congenital shunts, influencing strategic choices such as surgical or interventional procedures. This study compared the estimated shunt fraction (the ratio of pulmonary-to-systemic flow, Qp/Qs) for quantifying the left-to-right shunt in children with ventricular septal defect (VSD) using heart catheterization, four-dimensional (4D) flow, and two-dimensional (2D) flow magnetic resonance imaging (MRI). The goal was to establish a non-invasive and reliable measurement ratio between pulmonary and systemic blood flow in these patients.

**Methods:**

Between July 2022 and June 2023, patients scheduled to undergo invasive right heart catheterization were included in this study. MRI was performed one hour before the catheterization procedure. The correlation of shunt fraction was assessed between all methods after calculating the Qp/Qs ratio from 2D and 4D flow MRI and catheterization.

**Results:**

A total of 24 patients (aged 3–15 years, eight females) were ultimately included in the study. The Qp/Qs ratios obtained from 4D flow had a robust correlation (correlation coefficient r = 0.962) compared to those obtained during catheterization. Cardiac catheterization recorded the mean shunt fraction at 1.499 ± 0.396, while 4D flow measured it at 1.403 ± 0.344, with no significant difference between the two techniques. Moreover, there was a reasonable correlation (r = 0.894) between 2D flow measurements of Qp/Qs and the results obtained from catheterization, with a mean shunt fraction of 1.326 ± 0.283.

**Conclusion:**

4D flow MRI has the potential to be a non-invasive method for accurately measuring the left-to-right shunt in children with VSD.

## 1 Introduction

Congenital heart disease (CHD) affects about 1 in every 110 births, making it the most common congenital disorder (1–3). This condition encompasses functional problems and anatomical anomalies, including atypical dimensions, irregular connections, and structural abnormalities within the heart chambers, blood vessels, and adjacent veins. The physiological implications of CHD span a wide range, from asymptomatic cases detected only in adulthood to critical complications requiring immediate surgical intervention in infancy (4). Ventricular septal defect (VSD) is children's most common congenital heart defect and the second most frequent congenital anomaly in adults. VSD is a condition with communication between the right and left ventricles, leading to shunt formation, the primary mechanism of hemodynamic compromise. This process gives rise to pulmonary arterial hypertension (PAH), ventricular dysfunction, and an increased susceptibility to arrhythmias (5, 6). Nevertheless, remarkable progress in diagnosing, managing, and treating CHD has enhanced survival rates, leading to a growing population of individuals with CHD who are now reaching adulthood (7).

Measurement of the shunt fraction is crucial in guiding management and decisions for surgical or interventional approaches for patients with congenital shunts such as VSD (8, 9). Shunt quantification includes evaluating systemic flow (Qs) and pulmonary flow (Qp), which can be done through invasive or non-invasive techniques. An invasive approach for assessing pulmonary and systemic blood flow involves using right heart catheterization with oximetry. This method has long been recognized as the gold standard and allows calculating the shunt fraction using the Fick equation (10, 11). Transthoracic and transesophageal Doppler echocardiography and cardiac magnetic resonance imaging (MRI) constitute non-invasive approaches to assess flow. However, it is essential to note that there are limitations in quantifying shunt volumes through Doppler echocardiography. Reliable and reproducible Doppler shunt measurements depend on the presence of well-acoustic windows and the expertise of highly qualified operators, both of which are essential (12–14).

Cardiovascular evaluation with MRI is widely used to evaluate cardiovascular disease based on morphological and functional information. 2D (two-dimensional) planar phase-contrast cine imaging within cardiac MRI (PC-MRI) has been established as a reliable method for shunt quantification. However, as blood flow volume and velocity must be measured on a predetermined plane, the presence of an expert physician or highly qualified technologist during the scan is necessary (15, 16). Recently, the emergence of four-dimensional (4D) flow MRI has significantly advanced the field of flow imaging, enabling a thorough investigation of blood flow in arteries and the heart (17). Therefore, the application of this procedure has increased for visualization and quantification of blood flow in CHD patients (7). 4D flow MRI falls under the category of PC-MRI, involving three-dimensional (3D) anatomical coverage, velocity encoding along all three flow directions, and time-resolved relative to the dimension throughout the cardiac cycle. This modality allows for a comprehensive evaluation of intricate blood flow patterns by making it possible to visualize them in 3D and enabling adaptable retrospective quantification of flow parameters that can be performed in any plane within the acquisition volume (17–19).

While previous studies have compared the shunt fraction between invasive catheterization and 2D flow MRI in both childhood and adulthood (15, 16) and have directly compared 4D flow MRI and catheterization in adults (20, 21), there is a notable gap in comparing shunt fraction data from 4D flow MRI with catheterization in the pediatric age group. This gap is significant because effectively managing these patients during childhood is crucial. The objective of this study was to conduct a comparative analysis of shunt fraction measurements using 4D flow MRI and catheterization, concurrently with 2D flow MRI, in a specific pathology (VSD), focusing on patients with an average age of 10 years.

## 2 Materials and methods

### 2.1 Study plan

This prospective study was conducted at a single cardiovascular medical research center from July 2022 to June 2023. Thirty-one patients were recruited from those already scheduled for invasive right heart catheterization as part of their clinical care. Three patients were excluded due to claustrophobia, as we did not intend for patients to undergo general anesthesia (GA) for MRI. Two patients had their catheterizations canceled following consultation with an anesthesiologist for GA. Additionally, two patients were excluded due to technical problems with the MRI scan. Consequently, 24 patients remained in the study.

The primary objective was to compare shunt fraction measurements (Qp/Qs) between 4D flow MRI and right heart catheterization in patients. Concurrently, we aimed to perform a similar comparison using 2D flow MRI.

### 2.2 Cardiac MRI

All patients underwent cardiac MRI using a 1.5T Philips scanner [MR Systems Ingenia Ambition X, Release 5.7 2021-10-04 SRN = 47525 Nominal Main Magnetic Field (B0) = 1.5T, equipped with a 32-channel phased-array coil]. Cardiovascular magnetic resonance imaging (CMR) was performed without administering contrast agents or sedation, with retrospective electrocardiogram (ECG)-gating. It was scheduled one hour before catheterization over approximately 17–20 min, including the following scan items: [Q-4D flow, sQ-2D flow aorta, sQ-2D flow pulmonary and functional assessments of short axis (SA), left ventricle (LV), right ventricle (RV), two-chamber (2CH), three-chamber (3CH), and four-chamber (4CH)].

The analysis of 4D and 2D flow MRI was carried out after catheterization, following the guidelines provided by the software's owning company. Subsequently, a radiologist with over 10 years of expertise reevaluated the results (K.R-K).

### 2.3 4D flow MRI

**Figure 1:**
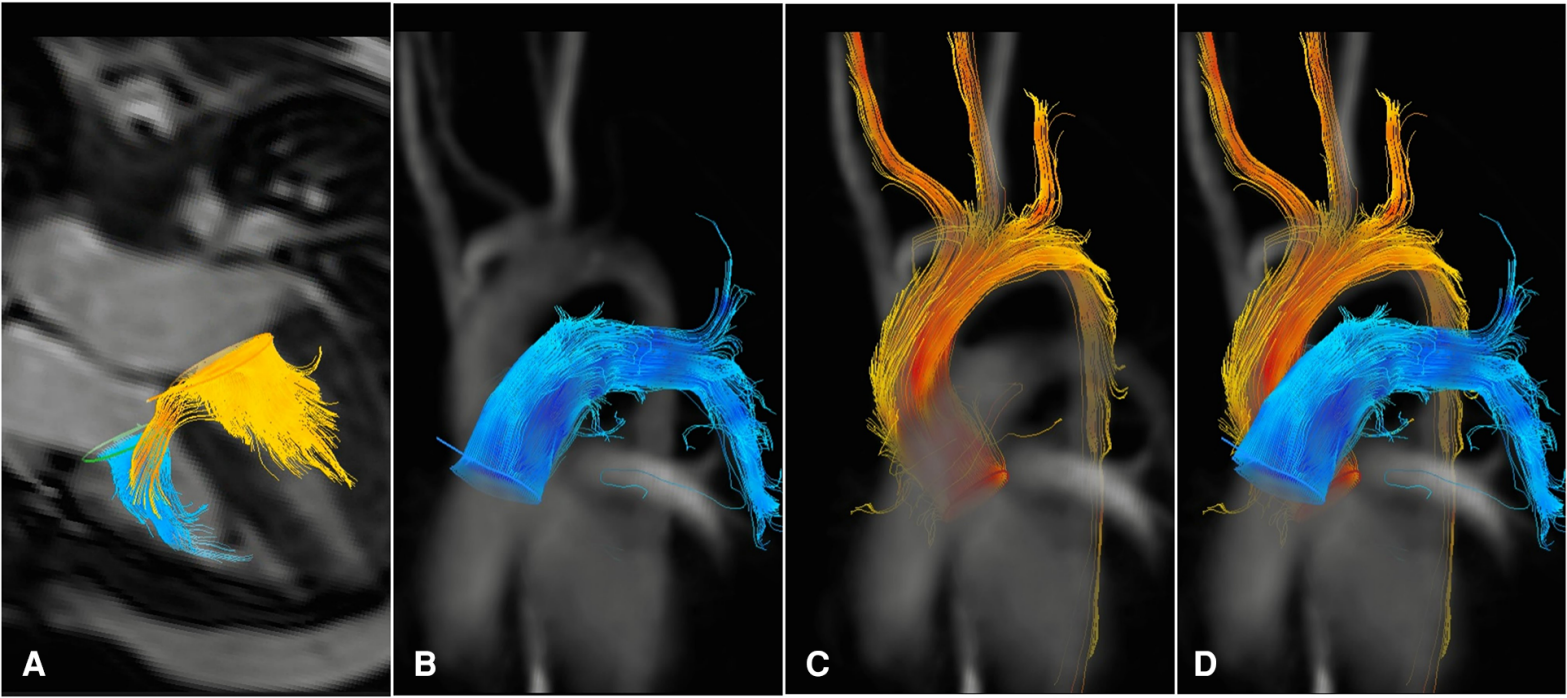
4d flow MRI images from a patient with VSD. The blood flow connection between the left and right ventricles can be visualized as yellow streamlines traversing through the ventricular septum during diastole (**A**) Blood flow measurements during systole are shown in the main pulmonary artery (**B**–**D**) and the ascending aorta (**C**–**D**).

4D flow was carried out using the same protocol for all patients, which included phase contrast without breath control (free-breathing) and compressed sensing, with each scan taking approximately 4–6 min. The average scan parameters were as follows: [Voxel Cor = 2.52 × 2.50 × 2.50; Rel SNR = 1.00; TE = 2.3; TR = 4.1—Geometry: FOV: FH = 333 mm, RL = 333 mm, AP = 110 mm; ACO voxel size: FH = 2.5 mm, RL = 2.5 mm, AP = 2.5 mm; Slice thickness = 2.5 mm; Recon voxel size: FH = 1.48 mm, RL = 1.48 mm, AP = 2.5 mm; Reconstruction matrix = 224; reduction = 8; slices = 44—Contrast: Contrast enhancement = T1; Flip angle = 8 deg—Dyn/Ang: Anglo/Contrast enhancement = phase contrast; Quantitative flow = yes; PC flow directions = RL-AP-FH; uniform velocity = yes; PC velocity = 150 cm/s]. Following previous research findings, for patients with confirmed or suspected venous shunting conditions, such as atrial septal defects (ASD) or VSD, a lower velocity encoding speed was recommended. In all our cases, this approach was adopted due to ventricular septal defects (VSD), necessitating a velocity-encoded value of 150 cm/s (20). The post-processing of data and calculation of the shunt fraction in 4D flow MRI were conducted using CAAS MR solutions software developed by Pie Medical Imaging company. Aliasing correction and window of interest with offset correction were employed as post-processing techniques. We used functional images to position a valve plane for visualizing and quantifying blood flow throughout the cardiac cycle. Functional LV and RV images were utilized for the aortic and pulmonary valves, respectively, while the 4CH view was employed for the mitral and tricuspid valves (Supplementary Figure S1). Blood flow was visualized by streamlines over the heart valves, which resulted from overlapping functional images on 4D flow (Figure 1). The results included forward and backward flow and the shunt fraction.

### 2.4 2D flow MRI

**Figure 2:**
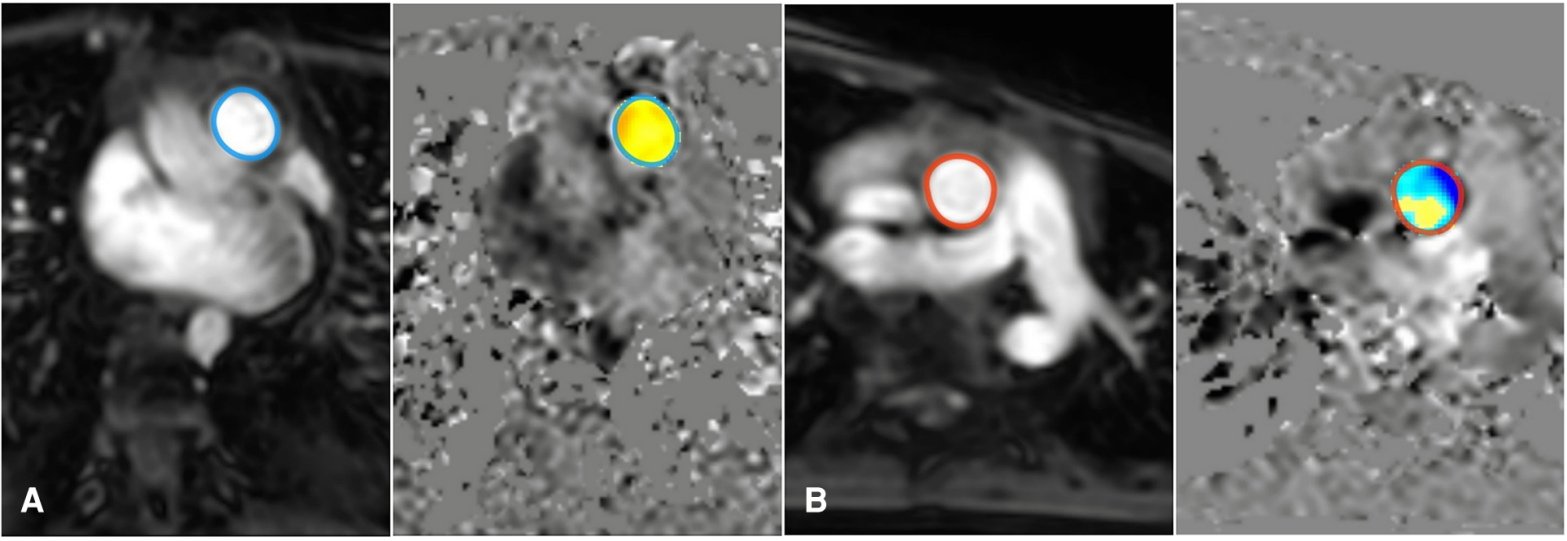
Measurement of the shunt fraction in 2D flow MRI: Add an ROI on the pulmonary artery (**A**), and ascending aorta artery (**B**).

Cardiac MRI with 2D planar phase-contrast cine imaging was performed using a retrospective gating technique on both the ascending aorta and the main pulmonary artery, with the following scan parameters:[Voxel Tra = 2.52 × 2.47 × 8.00; Rel SNR = 1.00; TE = 2.8; TR = 4.4—Geometry: FOV: RL = 262 mm, AP = 223 mm; ACQ voxel size: RL = 2.5 mm, AP = 2.5 mm; Slice thickness = 8 mm; Recon voxel size: RL = 1.09 mm, AP = 1.09 mm; Reconstruction matrix = 240—Contrast: Contrast enhancement = T1; Flip angle = 12 deg—Dyn/Ang: Anglo/Contrast enhancement = phase contrast; Quantitative flow = yes; PC flow directions = FH; PC velocity = 150 cm/s]. Slice position for aortic (Qs) and pulmonary (Qp) flow was approximately 2–3 cm distal to the aortic and pulmonary valves in the proximal ascending aorta and main pulmonary artery. Data post-processing in 2D flow MRI, akin to 4D flow, was accomplished using CAAS MR Solutions (Pie Medical Imaging) software. The calculation of the shunt fraction involved manual drawing of the cross-sectional areas of the pulmonary trunk and ascending aorta for each time frame on either the magnitude or phase images (Figure 2).

### 2.5 Right heart catheterization

**Figure 3:**
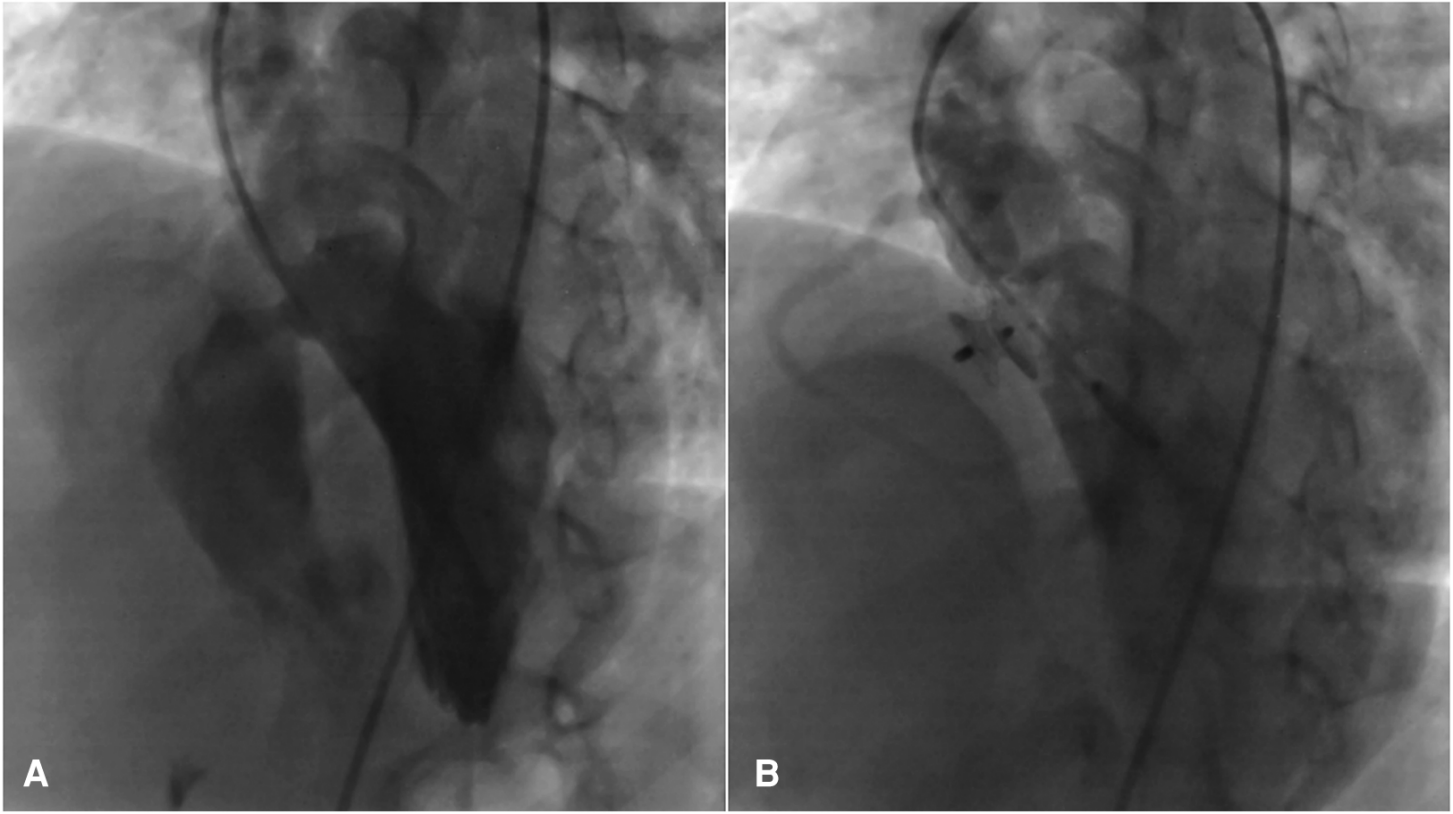
Significant peri membranous VSD extended to the sub-aortic septum, for which the 4D flow image was also displayed in (**A**), that was occluded by an 8 mm amplatzer asymmetric septal occluder (**B**).

Invasive oximetry was performed under sedation using the femoral vein method to obtain blood samples from the inferior vena cava (IVC), superior vena cava (SVC), right atrium (RA), right ventricle (RV), left ventricle (LV), pulmonary artery (PA), and ascending aorta (AO) before occluding the VSD (Figure 3). Subsequently, these blood samples were placed in an AVOXimeter device (a tool designed for measuring oxygen saturation) to measure oxygen saturation (SatO2). In the context of invasive oximetry, the shunt fraction was determined using the Fick equation as outlined below (22):

$$Qp/Qs=\frac{\mathrm{Ao}\;\mathrm{sat}\;\text{–}\;\mathrm{Mv}*\;\mathrm{sat}}{\mathrm{Pv}\;\mathrm{sat}\;\text{–}\;\mathrm{Pa}\;\mathrm{sat}}$$

$$*Mv=3\mathrm{SVC}\;\mathrm{sat}+\frac{IVC\;sat}{4}$$

Ao sat and Mv sat represent arterial and mixed venous oxygen saturation, while Pv sat and Pa sat indicate pulmonary venous and pulmonary arterial oxygen saturation. SVC sat and IVC sat refer to oxygen saturation in the superior vena cava and inferior vena cava, respectively.

### 2.6 Statistical analysis

The correlation between shunt fraction measurements obtained through 4D flow MRI analysis and catheterization data was evaluated using linear regression analysis with Pearson correlation, and their agreement was assessed using the Bland-Altman method. Statistical analyses, Bland-Altman plots, and scatter plots were performed using Prism GraphPad software. Similar statistical tests were conducted to compare Qp/Qs ratios derived from 2D flow data and catheterization, as well as between 4D and 2D flow (Supplementary Figure S2). A *P* value <0.05 was considered statistically significant for all analyses.

## 3 Results

### 3.1 Patients

Twenty-four patients were included in the final analysis. These patients had a mean age of ten years, with a standard deviation of ±4 years. Sixteen (67%) of the participants were male. All studied patients had a VSD with left-to-right shunt and no other cardiac abnormalities. Among them, 8 (33%) displayed Qp/Qs ratios lower than 1.3 (Qp/Qs<1.3), while 16 (67%) demonstrated ratios exceeding 1.3 (Qp/Qs>1.3). These assessments were based on catheterization data obtained using the Fick equation. Notably, among the entire cohort, diagnostic catheterization was performed without the need for immediate interventions in 4 cases. In contrast, VSDs were effectively occluded during the catheterization procedure in 15 cases using different types of VSD occluders, while 5 patients were subsequently referred for surgical treatment. Six patients were diagnosed with pulmonary hypertension, characterized by a pulmonary arterial pressure exceeding 20 mm Hg. Furthermore, eighteen individuals exhibited pulmonary pressures within the normal range, registering at 20 mmHg or below (Table 1).

**Table 1:** Patient characteristics.

Variables	Overall (*N* = 24)
Female	*N* = 8
Male	*N* = 16
Mean age (years)	10 ± 4
Height (cm)	136 ± 23
Weight (kg)	35 ± 21
Cardiac shunt	Left-to-right
Mean PAP (mm Hg) >20	*N* = 6
Mean PAP (mm Hg) ≤20	*N* = 18

Cardiac shunt and pulmonary artery pressure (PAP) were determined through a catheterization procedure.

### 3.2 Assessing shunt fraction: a comparative analysis of MRI and catheterization data

**Figure 4:**
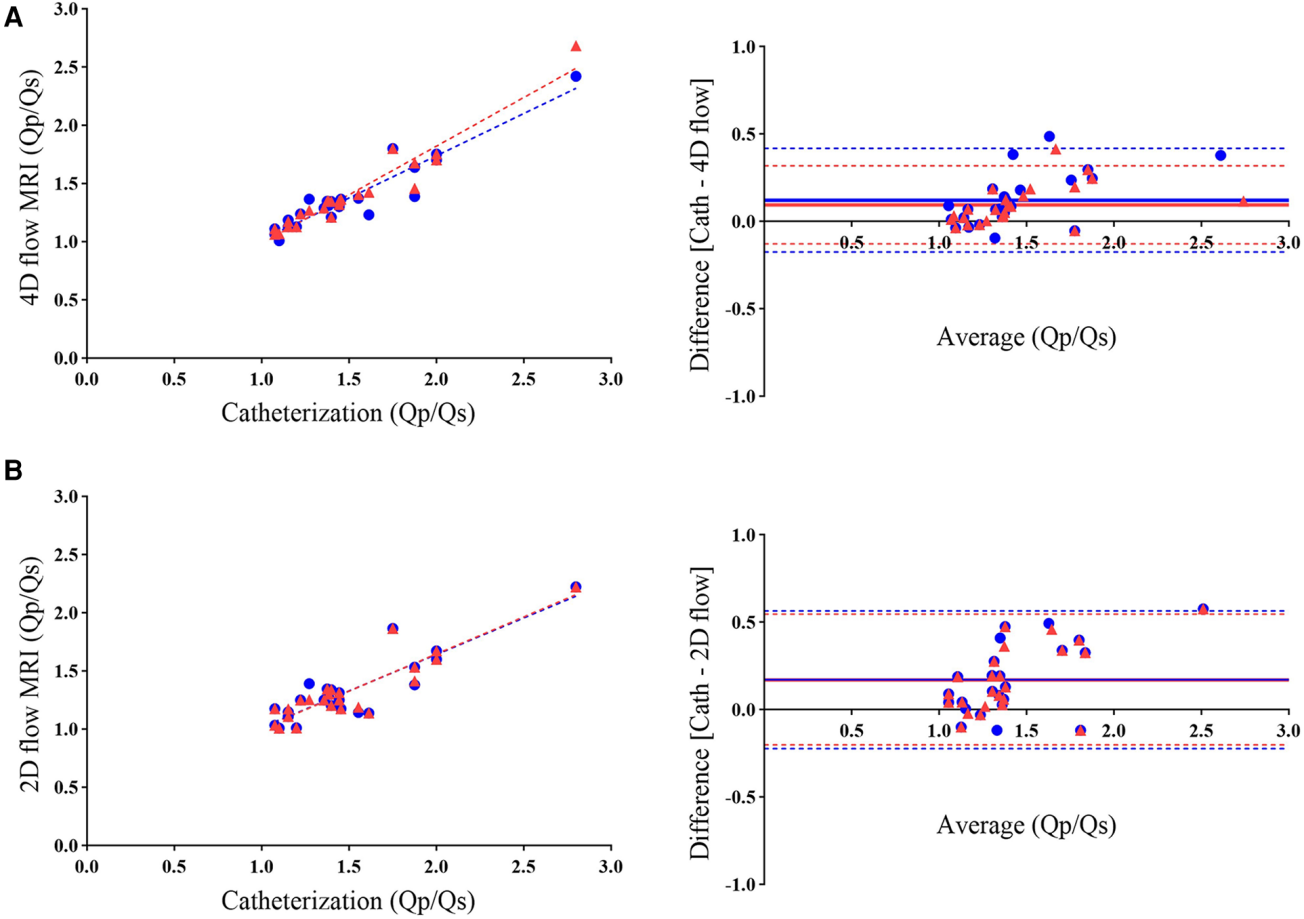
Scatterplots (left) and bland-altman analysis (right) illustrate the agreement between Qp/Qs measurements obtained using 4D flow MRI (**A**) and 2D flow MRI (**B**) with catheterization. The comparison reveals higher agreement in shunt fraction measurements with 4D flow than with 2D flow when referenced against catheterization. Blue circles represent initial data points from the analysis (R = 0.936; bias = 0.122), while red triangles indicate rechecked 4D flow measurements by an independent observer (R = 0.962; bias = 0.096). As mentioned in the manuscript, the overlapping of both colors in scatterplots and bias lines in Figure B demonstrates the lack of recheck ability in 2D flow.

The Qp/Qs ratios derived from 4D flow strongly correlated with those determined through catheterization (R = 0.962; 95% CI: 0.9133–0.9838; *P* < 0.0001). The mean shunt fraction was 1.403 ± 0.344 when evaluated using 4D flow, while it measured 1.499 ± 0.396 through cardiac catheterization, revealing no significant difference between the two methods. The bias was 0.096, and the limits of agreement ranged from −0.127 to 0.319 (Table 2). Bland-Altman plots also displayed strong agreement between the two methods (Figure 4). In 21% of the patients, the shunt fraction obtained from 4D and 2D flow exceeded the values observed in catheterization. In contrast, 79% of the cases demonstrated lower values when compared to the shunt fraction obtained through catheterization.

**Table 2:** Comparison of shunt fraction 4D flow MRI, catheterization, and 2D MRI.

Parameters Procedures	Pearson correlation coefficient	Mean difference (point value)	Upper limit	Lower limit
4D flow vs. catheterization	R = 0.962	0.096	0.319	−0.127
2D flow vs. catheterization	R = 0.894	0.172	0.546	−0.201
4D flow vs. 2D flow MRI	R = 0.969	0.049	0.199	−0.099

The mean difference (point value) and the upper and lower limits were derived from the Bland-Altman (BA) analysis. The correlation coefficient (R) was obtained using the Pearson method.

Analysis of 2D flow yielded Qp/Qs ratios that exhibited a fair correlation with those calculated through catheterization using the Fick equation (R = 0.894; 95% CI: 0.7687 to 0.9538; *P* < 0.0001). The mean shunt fraction was 1.326 ± 0.283 when measured through 2D flow. At the same time, the Bland-Altman analysis indicated excellent agreement between the Qp/Qs ratios obtained through 2D flow and catheterization; this level of correlation was lower than the results obtained between 4D flow and catheterization (Figure 4 and Table 2).

## 4 Discussion

For effective management and choosing the best treatment for patients with cardiac shunts, accurate measurement of shunt severity is imperative (23–29). While the conventional approach often involves invasive catheterization, this study advocates the utilization of 4D flow as a non-invasive alternative for assessing shunt-related issues or as a valuable preliminary step in the assessment process before considering catheterization. Furthermore, it suggests that invasive catheterization can be postponed in specific cases until additional information about the heart's structure or function is required to conduct a more comprehensive evaluation. Data obtained through invasive oximetry for Qp/Qs ratios (shunt fraction) demonstrated high consistency and correlation when employing 2D and 4D flow MRI techniques. Despite the time-consuming nature of 4D flow compared to 2D flow in terms of data acquisition and measurement of hemodynamic parameters, the Qp/Qs ratio obtained from invasive catheterization aligned more consistently with the results from 4D flow MRI than those from 2D phase contrast. Additionally, 4D flow MRI offers other advantages, such as comprehensive anatomical assessment and the potential for future post-processing analysis of evolving hemodynamic indices. Moreover, respiratory motion does not adversely affect measurement accuracy in 4D flow MRI, thanks to advanced techniques like compressed sensing and free-breathing protocols (30, 31). Therefore, our research demonstrates that measuring pediatric left-to-right shunts using 4D flow is feasible and reliable. Consequently, in some cases, 4D flow can replace invasive testing, resulting in cost savings, reduced patient discomfort, and the prevention of severe but uncommon consequences associated with catheterization.

Our study specifically focused on calculating the shunt fraction from pulmonary and systemic flow. This emphasis stemmed from previous research, which indicated that the Qp/Qs ratio obtained from blood flow measurements is more accurate in clinical settings than ventricular volumetry (20).

Hemodynamic assessment through cardiac catheterization is a valuable diagnostic tool for evaluating various cardiovascular conditions. However, it is not without its complexities and potential complications. These complications encompass ventricular arrhythmias, temporary right bundle branch block, and complete heart block. Additionally, air embolism is risky if air enters the catheters or pressure transducers, leading to sudden chest pain, dyspnea, and hypotension. Pulmonary artery perforation, although infrequent, can occur during extended catheter placement, especially in patients with prior pulmonary hypertension and those undergoing anticoagulation therapy (32, 33). Indwelling pulmonary artery catheters are associated with various potential issues, including infections, pulmonary infarctions, perforations, and arrhythmias. Furthermore, there is a risk of allergic reactions to contrast agents and radiation exposure during x-ray fluoroscopy. Vein thrombosis is another potential complication. Additionally, sedation, agitation, and breathing issues can negatively impact venous and arterial blood oxygenation. Nevertheless, a recent study found no difference in Qp/Qs regardless of the sedation method employed, although it did note lower values for PAP and PVRI under general anesthesia (34). It is important to note that genuine mixed venous saturation is computed rather than directly measured by taking upper and lower vena cava samples (35, 36). Given these drawbacks, discussing the continued use of invasive oximetry as the gold standard compared to non-invasive techniques is imperative.

Addressing the complexities of MRI scans and the limitations that existed in our study is very important. Conducting MRI scans in pediatric patients presents unique challenges, as anatomical structures are minor, necessitating higher spatial resolution. Additionally, pediatric patients often have higher heart rates, demanding superior temporal resolution to mitigate motion artifacts. Some patients may require sedation due to uncooperative behavior and claustrophobia (37, 38). Moreover, it's essential to note that this study was conducted for research purposes, and there was no significant time delay between imaging and invasive catheterization in all cases. However, if a delay were to occur between these two methods, it could potentially impact the agreement between catheterization and 4D flow measurements, possibly due to changes in medication or other environmental conditions. Furthermore, the study was conducted at a single location using a single MRI scanner, with the same technologist, protocols, and software for analysis. The successful application of the more recent 4D flow technique requires a certain level of expertise, including a comprehensive understanding of potential imaging challenges, such as errors related to vortical flow and the identification of various artifacts, including those arising from turbulence, dephasing and aliasing (39, 40). Despite these complications and the ethical imperative that prohibited the use of contrast agents and anesthesia during cardiac imaging, we observed a marginally higher correlation between the shunt fraction obtained from 4D flow MRI and catheterization compared to the study conducted in adults, where contrast agents were used in CMR (20, 21). Therefore, the present study demonstrates that 4D flow MRI in pediatrics, even without contrast media, could feasibly measure the left-to-right shunt in children with VSD using standard scan settings (18).

In conclusion, this study underscores the potential of 4D flow MRI as a non-invasive method for measuring left-to-right shunts in children with VSD, providing a comparable alternative to invasive cardiac catheterization with oximetry for these patients. These findings support the use of Qp/Qs results obtained through 4D flow MRI in assessing and managing such patients.

## Data Availability

The raw data supporting the conclusions of this article will be made available by the authors, without undue reservation.
