# Supplementary material for: Assessment of 4D flow MRI for quantification of left-to-right shunt in pediatric patients with ventricular septal defect: comparison with right heart catheterization
Source: Front Cardiovasc Med. 2024 Jul 22;11:1399110. doi: 10.3389/fcvm.2024.1399110 (PMC11298441; doi:10.3389/fcvm.2024.1399110)
Supplement: Supplementary file 7 [file Datasheet1.docx]

Supplementary Material

# Supplementary Figures


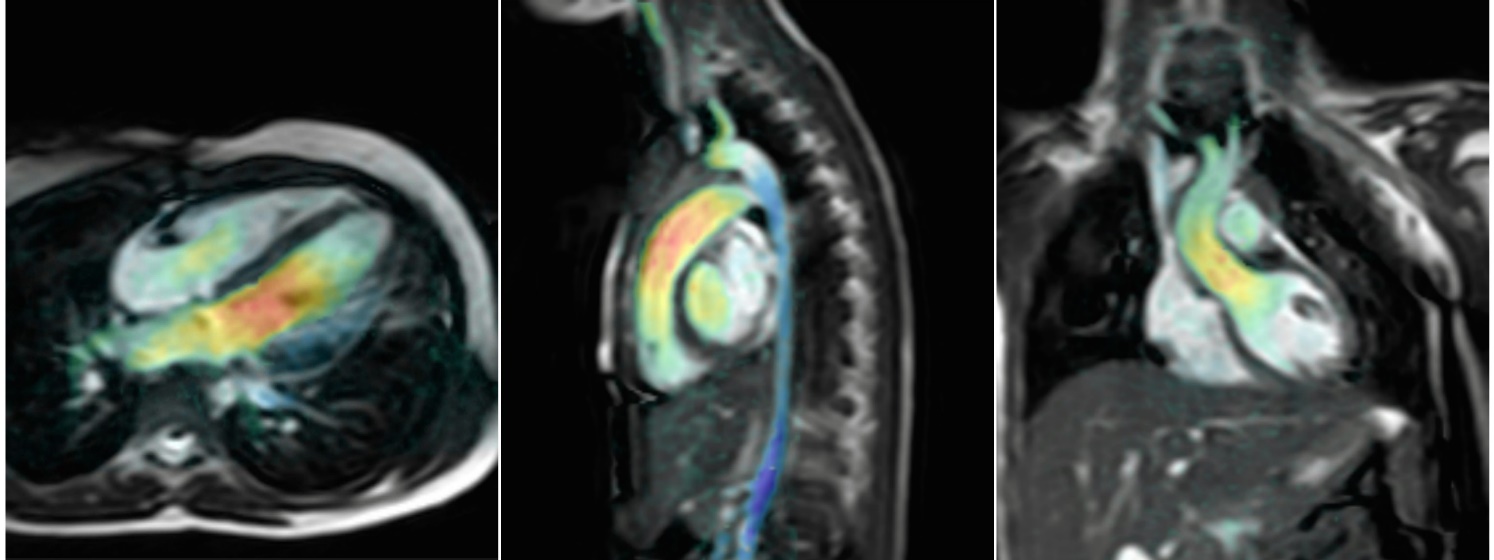


C

A

B

**Supplementary Figure S1.**: We used functional images to position a valve plane to visualize and quantify blood flow throughout the cardiac cycle. Functional LV (C), and RV (B), images were utilized for the aortic and pulmonary valves, respectively, while the 4CH (A), view was employed for the mitral and tricuspid valves.

## Supplementary Figures


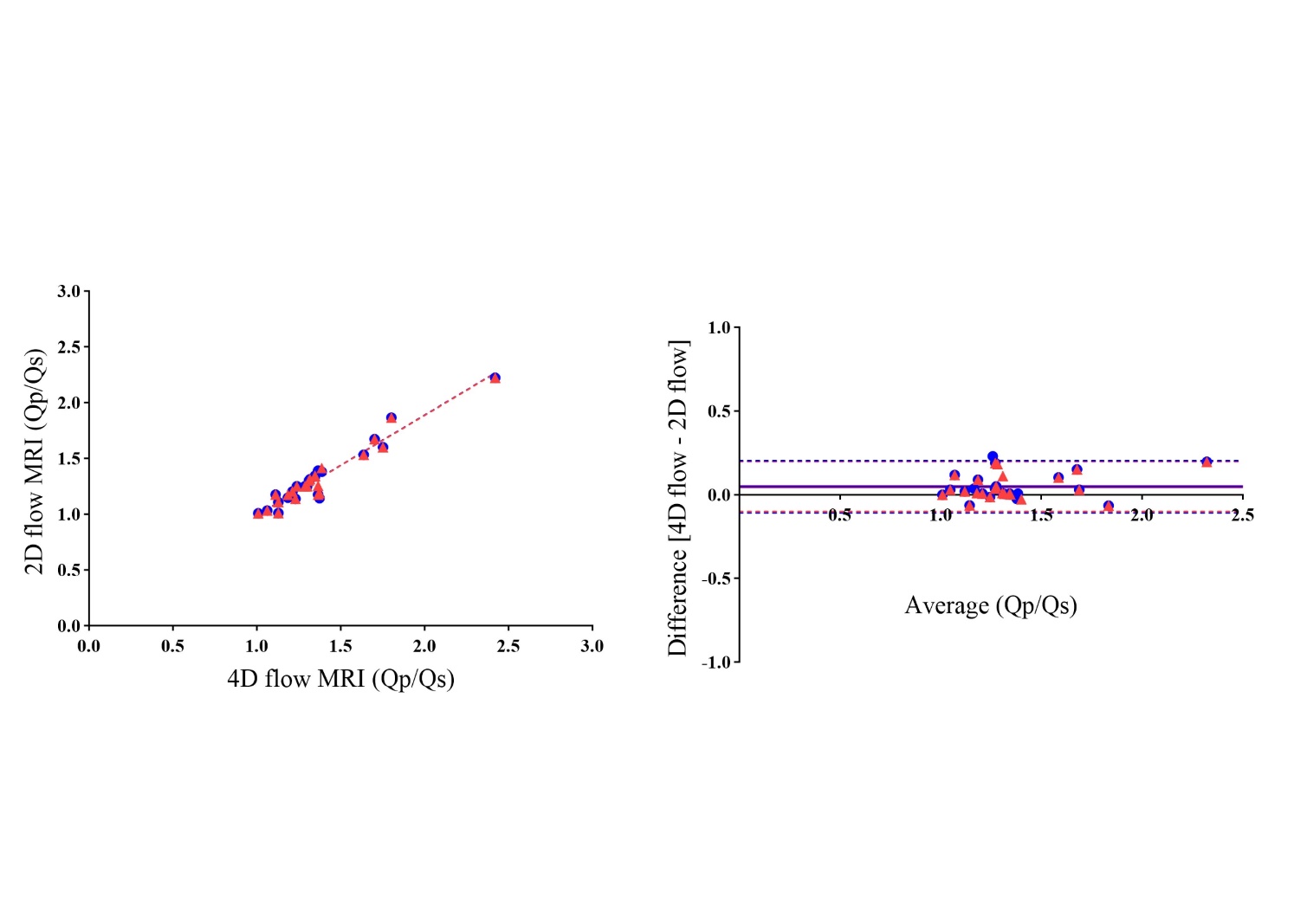


**Supplementary Figure S2.**: Comparison of systemic (Qs) and pulmonary (Qp) blood flow measurements by four-dimensional (4D) flow MRI and two-dimensional (2D). Bland-Altman analysis (left) and scatterplots (right) indicate higher agreement between shunt fraction measurements with 4D flow (A) with 2D flow (B). Blue circles represent initial data points from the analysis, while red triangles indicate rechecked 4D flow measurements by an independent observer.
